# Short-Term Outcomes of Interdisciplinary Hip Fracture Rehabilitation in Frail Elderly Inpatients

**DOI:** 10.1155/2018/1708272

**Published:** 2018-12-31

**Authors:** Manuel Bayon-Calatayud, Ana Maria Benavente-Valdepeñas

**Affiliations:** Department of Physical and Rehabilitation Medicine, Complejo Hospitalario de Toledo, Servicio de Salud de Castilla-La Mancha (SESCAM), Avenida de Barber, 30, ES- 45004- Toledo, Spain

## Abstract

**Objective:**

To investigate short-term outcomes of an interdisciplinary rehabilitation program for elderly inpatients who underwent surgical treatment for hip fractures.

**Methods:**

This is a prospective cohort study of fifty older inpatients who were admitted to a geriatric rehabilitation unit. Clinical and functional outcomes were assessed at admission, at discharge, and one month postdischarge.

**Results:**

Patients mean age was 84.1 ± 4.7 years. Proportions of study population with risk factors of frailty were cognitive impairment (64%), Charlson comorbidity index > 1 (72%), and protein malnutrition (59.2%). Before fracture, Barthel median was 90 (IQR 85, 100), and functional ambulation classification (FAC) score was ≥ 4 for 90% of study participants. One month after concluding rehabilitation, Barthel median was 80, 1 month postdischarge FAC ≥ 4 – prefracture FAC ≥ 4 mean change was – 8% (95% CI, -21.5%, 3.4%), and average for gait speed was 0.48 ± 0.18 m/s (95% CI, 0.43, 0.54). Significant correlation was found between admission Barthel score and 1 month postdischarge Barthel score (*ρ*= 0.27, p=0.05), and between prefracture FAC score and FAC score 1 month postdischarge (*ρ* = 0.57, p = 0.05). According to regression analysis, age, cognitive status, prefracture Barthel, prefracture FAC, type of surgery, and length of stay were associated with short-term recovery outcomes.

**Conclusion:**

An early interdisciplinary rehabilitation management was insufficient to recover prefracture functional status. Future studies should investigate the best therapeutic strategies to optimize functional recovery, according to clinical and prefracture frail conditions of these patients.

## 1. Introduction

Hip fracture is an important health problem because of its associated morbidity and high economic costs for healthcare providers. This fracture is particularly predominant in older populations with a 3:1 women/men ratio. In the United States in 2010 there were 258,000 hospital admissions for hip fracture among people aged 65 years and older [1]. Bone loss due to osteoporosis, muscle atrophy, lack of motor coordination, and cognitive impairment are factors that could facilitate falls, which eventually may cause a hip fracture.

The majority of these patients undergo a surgical intervention. Hip fracture surgical management depends on fracture location, age, and patient condition. Patients with displaced intracapsular hip fracture could be treated by total hip replacement or, alternatively, by cemented hip hemiarthroplasty. Extracapsular fractures could be treated by open reduction and internal fixation. Intramedullary nails are preferred in case of intertrochanteric fractures. Complications after hip fracture surgery are common due to poor vascularization of femoral neck, or related to mechanical factors and load bearing [2].

Traditionally, in our country these patients stayed at acute hospital for several days (5-7 days). In absence of complications, they were discharged and referred to home where they usually awaited for several days or weeks until they could start functional recovery at an outpatient centre, where rehabilitation was focused on returning patients to their prefracture functional status.

During the last 25 years, a novel strategy for postoperative care of these patients was gradually implemented. This therapeutic strategy consisted in early discharge from acute hospital to a rehabilitation ward in a geriatric hospital. These geriatric rehabilitation units usually were integrated by a coordinated medical team including traumatologists, geriatricians, and rehabilitation physicians.

Although there is a tendency to find better results for this care pathway, there is still lack of evidence from randomized trials to establish the best strategies for enhancing mobility after hip fracture surgery [3–5].

A frailty state is not unusual among older people with hip fracture [6]. Frailty was defined by the occurrence of at least 3 of the following deficits in an individual: slow walking speed, impaired grip strength, a self-report of declining activity levels, unintended weight loss, or exhaustion [7].

Increasing levels of frailty before hip fracture and short-term hospitalization [8, 9] may affect functional recovery even after an appropriate rehabilitation management.

The purpose of this study was to analyse prefracture status and short-term functional outcomes achieved by frail elderly inpatients with hip fracture who followed an early interdisciplinary rehabilitation program.

## 2. Material and Methods

### 2.1. Study Design

This is a prospective cohort study. It was performed according to Helsinki declaration, and it has been approved by the Research and Ethics Committee from Complejo Hospitalario de Toledo.

### 2.2. Participants

**Figure 1:**
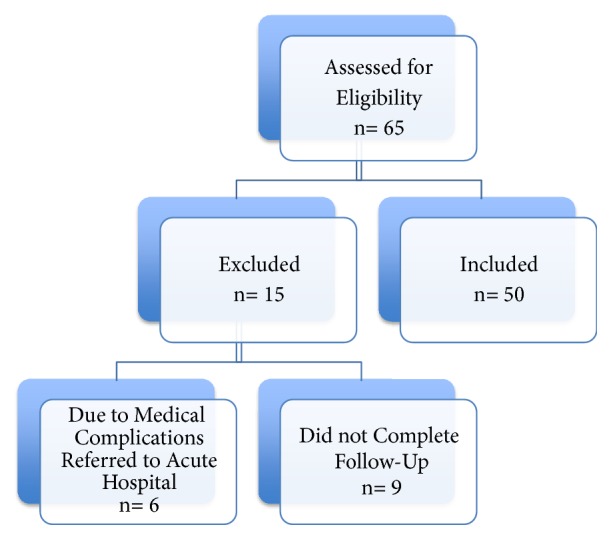
Flow diagram of study patients.

A cohort of 50 inpatients who underwent hip fracture surgery were recruited from April 2016 to June 2017. All of them met inclusion criteria and were admitted and included during this period. Patients flow diagram is shown in Figure 1.

Written informed consent was provided to patients. Information about the procedure and objectives of the study were also given to patients, relatives, and caregivers.

Postoperatively, patients were transferred from acute care hospital and admitted to a geriatric rehabilitation ward of a university-affiliated referral geriatrics hospital.

Inclusion criteria for study patients were as follows: patients who followed hip fracture surgery (hip replacement arthroplasty, or internal fixation by intramedullary nail), with stable medical condition, without weight-bearing restrictions, and were enabled to perform an active rehabilitation treatment.

Exclusion criteria were as follows: patients with any kind of unstable medical condition, severe cognitive impairment (Mini–Mental State Examination- MMSE score ≤ 12), hemiparesis, neoplasic hip fracture, prior contralateral hip fracture, open hip fracture, or other concomitant lower limb orthopaedic disorders. Patients who were simultaneously involved in other rehabilitation studies were also excluded.

### 2.3. Clinical Assessment

Prefracture functional status, patients characteristics, and other data of interest (age, sex, type of residence, type of fracture, surgical procedure, and time passed since surgery) were collected from medical records and through interview with patients, relatives, and caregivers. Prior comorbidities and cognitive status were assessed by using Charlson comorbidity index (CCI) and MMSE, respectively. Furthermore, nutritional status was measured by MiniNutritional Assessment (MNA) scale and albumin serum blood levels.

During the first medical visit at ward, a functional status evaluation was made by rehabilitation physicians who implemented an individualised rehabilitation program considering prefracture clinical and functional status. Walking ability was assessed by using functional ambulation classification (FAC). In addition, aids for walking and ability to climb stairs were assessed through a scale rated as 5= no aids for walking, able to climb stairs, 4= one cane, able to climb stairs, 3= two canes, able to climb stairs, 2= walker assistance, able to climb stairs, 1= walker assistance, unable to climb stairs, and 0= unable to walk.

Clinical assessment was focused on identifying and treating comorbidities and postoperative complications. Blood samples pickup, electrocardiogram, temperature, pulse, oxygenation, and blood pressure measurements were carried out by geriatric nursing team. Patients received 40 mg of a low weight molecular heparin daily postoperatively at least during 21 days. Prophylactic antibiotics, calcium, and supplements of vitamin D were usually prescribed after surgery. Routinely analgesics were provided for pain relief. Decubitus ulcers prophylaxis was made by pressure relieving mattresses. Blood transfusion was ordered if hemoglobin was lower than 10 g/dl, and oxygen supply was provided if saturation was lower than 95%.

Last day of stay at geriatric rehabilitation unit, and before being discharged, patients were assessed by measuring functional and cognitive variables (Barthel, Montebello index, FAC, MMSE). Other data collected at this time were length of stay at geriatric rehabilitation unit and the final destination of patients (own home, nursing home).

A Barthel score ≥ 60 and ability for safe walking (FAC ≥ 2) were considered as criteria for discharge.

One month after being discharged evaluations of functional independence, cognitive status, and walking ability were made, including measurement of gait speed in 6 meters walking aisle. An index for the change on functional recovery regarding prefracture functional status was also calculated (1 month postdischarge Barthel – admission Barthel / prefracture Barthel – admission Barthel).

### 2.4. Rehabilitation Management

Rehabilitation consisted of a daily 60-minute individualised program.

During the first week, physiotherapy was based on isometric muscle strengthening of lower limbs, lower limbs range of motion exercises with flexion of affected hip limited to 90°-100°, abduction limited to 0-30°, avoiding adduction and rotation movements of operated hip. Simultaneously, an occupational therapy program was implemented based on transfer training, instruction for performing activities of daily living, and technical aids assessment.

During the second week, isotonic muscle strengthening, transfer training and balance exercises within parallel bars were implemented. Finally, during the third week, patients began a functional gait training (parallel bars, walker, crutches, progressively climbing stairs).

Patients were daily examined and assessed at ward by nursery, physical therapists, geriatricians, and rehabilitation physicians. A social worker made an evaluation of patients' needs regarding social support, providing information about social services and community resources.

Weekly interdisciplinary meetings were implemented to follow up recovery process and for planning hospital discharge according to patient final destination and available social support.

### 2.5. Statistical Analysis

Statistical analysis was performed using SPSS 17.0 software package (SPSS Inc., Chicago, IL, USA). Values for continuous variables were reported as means and standard deviation (SD). Qualitative and ordinal variables were expressed as percentages, medians, and interquartile ranges. Correlations between variables were studied by Spearman correlation test. 95% confidence intervals (CI) were calculated for studied variables. Logistic regression analysis and backward multivariate regression analysis were made in order to study association between variables and clinical outcomes. Statistical significance was accepted for p-values less than 0.05.

## 3. Results

Demographic and clinical characteristics of study patients are shown in Table 1.

Mean age for study patients was 84.1 ± 4.7 years; 78% of them were women (95% CI 64.8, 87.2). Intracapsular fractures were more frequent among women (71.8%) than men (63.6%). In contrast, extracapsular fractures were more frequent in men (36.4%) than women (28.2%).

Since surgery until beginning of rehabilitation mean time passed was 6.4 ± 2 days. Length of stay average at rehabilitation ward was 21.9 ± 6.1 days.

Before hip fracture, Barthel median was 90 (IQR 85, 100), and FAC score was ≥ 4 for 90% of study participants.

Barthel median at admission to the rehabilitation unit was 35 (IQR 20, 40), cognitive impairment (MMSE < 24) was presented by 64% of patients with hip fracture, Charlson comorbidity index (CCI) ≥ 2 was found for 48%, and low albumin blood serum levels (<3.5 g/dl) were detected for 59.2% of them (95% CI, 45%, 72%).

At discharge, after concluding rehabilitation, Barthel median was 70 (IQR, 65, 85) and Montebello index mean score (functional recovery regarding prefracture functional status) was 0.7 (95% CI 0.66, 0.77) with 1 being the maximum index score.

One month after rehabilitation discharge, Barthel median was 80, and functional recovery regarding prefracture status was 0.84 (95% CI, 0.77, 0.90). At this time FAC score ≥ 4 was presented by 82% of study patients with a 1 month postdischarge FAC ≥ 4 – prefracture FAC ≥ 4 change of – 8% (95% CI, -21.5%, 3.4%). In addition, at least one degree of ambulation on FAC scale was lost by 18% of study participants (95% CI, 9.8%, 30.8%).

Before hip fracture 36% of patients were able to walk independently without walker or canes (95% CI, 22%, 50%). One month after being discharged, 54% of patients still needed one cane for walking, 6% two canes, and 32% a walker. Proportion of patients who were able to climb stairs decreased by 10% (1 month postdischarge stairs – prefracture stairs change -10%; 95% CI, 23.9%, 4.1%). Mean value for gait speed, assessed 30 days after hospital discharge, was 0.48 ± 0.18 m/s (95% CI, 0.43, 0.54). Most of them (75%) had a gait speed lower than 0.6 m/s.

Significant correlation was found between Barthel score at admission and Barthel score 1 month postdischarge (*ρ*= 0.27, p=0.05), and between prefracture FAC score and FAC score 1 month postdischarge ((*ρ* = 0.57, p = 0.05).

According to regression analysis outcome (Tables 2 and 3), age, cognitive status, prefracture walking ability, functional independence prior to hip fracture, and length of stay at rehabilitation unit were variables associated with recovery of walking and functional independence 1 month postdischarge. Surgical management by intramedullary nail appears to be associated with short-term ambulation loss experienced by patients from this cohort.

## 4. Discussion

Hip fracture is one of the most common traumatic events which may occur among elderly people. The mean age for this cohort was 84 years old, older than in other recent studies [10, 11]. Older age did not seem to be a decisive factor to affect prefracture functional independence of patients (prefracture Barthel median 90). However, after hip fracture surgery, a significant change occurred in patients condition and older age emerged as a determining factor for recovery. In fact, older age was found associated with short-term ambulation loss and worse functional recovery outcomes, for patients of this cohort.

Hip fracture was predominant among women (78%), probably due to longer life expectancy compared to men. Similarly to prior studies [12, 13], sex was not found as a variable that could affect functional recovery outcomes.

Patients of this study had adequate social support provided by their families and caregivers. One month after finishing rehabilitation, only 14% of study patients were living at a nursing home. This proportion was lower than that reported by other authors [14]. Despite this good social support, patients did not recover prefracture functional status at short-term after being discharged.

In this research, similarly to what was reported by prior studies [15, 16], extracapsular hip fractures were associated with poor functional recovery outcomes. It could be related to older age, osteoporosis, and more frequent load-bearing complications which may delay rehabilitation and recovery process.

Comorbid conditions may also have a negative impact on functional recovery after hip fracture. Leibson et al. [17] reported that 45% of hip fracture patients had a CCI >1. For patients of this study this proportion was even greater (72% had a prefracture CCI > 1, and CCI ≥ 2 was found for 48%). A great comorbid disease burden at the time of the fracture could be a marker of physical frailty, and it may be associated with worse short-term recovery outcomes.

Cognitive function, nutritional status, and preinjury functional level are three main factors closely related to hip fracture rehabilitation success [18].

Cognitive impairment is a contributing factor to a frailty state that may influence rehabilitation outcomes. In this research, at the time of admission to rehabilitation unit, cognitive impairment (MMSE < 24) was presented by 64% of study patients. This proportion was greater than 42% estimated prevalence by some authors [19].

It has been calculated that 50% of subjects with impaired cognition required human assistance for walking [20]^.^ In this study, one month after finishing rehabilitation 92% of patients still needed assistance for walking (canes or walker).

This study showed association between cognitive status and short-term functional recovery outcomes. Perhaps frail older populations with severe cognitive impairment could not benefit enough from intensive interdisciplinary rehabilitation programs. Their performance could be worse and undergraded compared to patients cognitively intact. However, it is very questionable to exclude these patients of rehabilitation programs, because they still may achieve functional gains from rehabilitation [21].

Malnutrition has been associated with poor functional recovery, with increased requirements regarding walking aids, and longer length of stay [22]. The prevalence of malnutrition in elderly hip fracture patients ranges between 52% and 64% [23].

Although for this cohort 59.2% of patients presented low blood serum levels of albumin (<3.5 gr/dl), no significant association with short-term functional outcomes was found (p= 0.083). This could be explained by the fact that most of these patients had a mild decrease of blood serum proteins, with albumin mean levels (3.37 ± 0.46) close to normal.

Prefracture functional status is another main predictive factor of recovering after hip fracture surgery [24]. In this research, prefracture Barthel was associated with functional gains 1 month postdischarge.

Some authors reported that three months after hip fracture, 34%-59% of patients achieved the same level of functional independence on ADLs performance as they had before fracture [25]. In the present study, functional independence in terms of no need for assistance, but requiring more time for ADLs performance, was achieved by 46% of patients. This proportion was 62% before hip fracture. It is evident that patients of this cohort, even after following an early intensive rehabilitation program, did not recover prefracture functional level.

Proportion of study participants who achieved short-term independent walking (FAC ≥ 4) was 82%. Although it was greater than that reported by a prior similar study (55.4%) [26], patients did not achieved the same ability of walking as before fracture (FAC ≥ 4 = 90%).

One study showed that 14% of hip fracture patients were able to walk without aids at three months after being discharged [27]. In this study a reduced proportion of 8% among study patients were able to walk independently without aids 1 month postdischarge. It has been estimated that gait speed should be approximately 1-1.2 m/s for a safe outdoor walking [28]. A low gait speed (<0.8 m/s) may suggest a poor muscle function and sarcopenia [29].

In this research, 1 month after being discharged most of patients (75%) achieved a gait speed lower than 0.6 m/s, insufficient to allow a safe outdoor walking.

Before hip fracture, most of patients had a good prefracture functional status. Nevertheless, they also had risk factors of frailty such as older age, great comorbidity, cognitive impairment, and protein malnutrition. Hip fracture induced on this predisposed population a real frailty state that may have influenced rehabilitation outcomes.

Early intensive rehabilitation has been recommended after hip fracture surgery to prevent postoperative complications and to achieve an early recovery of functional mobility [30]. Patients of this study started early acute rehabilitation 48 hours postsurgery. Rehabilitation was continued until admission to geriatric rehabilitation unit, where they began the second phase of recovery treatment. Although an early intensive rehabilitation program was implemented, the patients of this cohort were not able to recover prefracture functional independence and required more assistance for walking than before hip fracture. Moreover, recovery of gait speed was insufficient and it was probably influenced by poor muscle function and sarcopenia. Nevertheless functional short-term improvements achieved after discharge may be considered a favourable result and a success derived from the intensive rehabilitation program implemented.

Although some criteria for discharge (Barthel ≥ 60, FAC ≥ 2) were used from the beginning of the study, often these criteria were patient-focused depending upon patients' needs (i.e., indoor or outdoor ambulatory, independence on stairs), making more emphasis on these goals or needs than on objective measures of performance (walked distance, gait speed, and walking aids). So this fact potentially could have affected length of stay, treatment duration, and even functional gains 1 month postdischarge.

After early recovery phase, possibly it could be useful to implement an outpatient individualised rehabilitation program based on muscle strengthening with resistance exercises in order to maximise functional recovery and to prevent the development of sarcopenia [31]. However, many frail patients with hip fracture may have severe cardiovascular comorbidities so they could have problems to be eligible for this kind of exercise program, and the issue for optimizing postoperative functional recovery in these patients still remains to be solved.

### 4.1. Study Limitations

Study limitations may include the fact that participants of this study came from a single geriatric rehabilitation unit. Only a short-term follow-up was made (1 month postdischarge). A 3-month postdischarge follow-up could be of interest for future studies in order to complete and compare 1-month to 3-month short-term functional outcomes. At admission into the unit, prefracture functional status was assessed based on retrospective information provided by relatives, caregivers, and patient recall. Several studies consider that this could be a valid and acceptable way to obtain functional and clinical information [32, 33].

## 5. Conclusions

It seems to be crucial to identify frailty in elderly populations prone to suffer from a hip fracture. Frailty may be a potential predictor of short-term recovery outcomes, having important implications for functional prognosis and even for planning rehabilitation interventions.

Future controlled and long-term studies should compare rehabilitation outcomes of frail and not frail populations. They should also investigate the best therapeutic strategies according to clinical and prefracture frail conditions of patients with hip fracture.

## Figures and Tables

**Table 1:** Characteristics of study population.

Demographic and clinical characteristics

(i) N (male/female) 50 (11/ 39). Mean Age ± SD: 84. 1 ± 4.7 (73- 95)
(ii) Living arrangement before hip fracture Family home 66 %
Partner- at own home 24 %
Caregiver- at own home 8 %
Nursing home 2 %

(iii) Fracture type/Surgery: Intracapsular- Hemiarthroplasty 70 %
Extracapsular- Intramedullary Nail 30 %
(iv) Delay mean time until rehabilitation (days) 6.4 ± 2
(v) Pre-fracture CCI: 48% CCI ≥ 2
(vi) MMSE-admission mean ± SD: 21.9 ± 4.5 (14- 30). 64 % MMSE < 24.
(vii) MNA median(IQR)- admission: 12 (9, 13).
(viii) Albumin serum level- admission, mean ± SD: 3.36 ± 0.46
(ix) Barthel median (IQR)
*pre-fracture* 90 (85, 100)
*1 month post-discharge* 80 (68.75, 90).

(x) FAC ≥ 4
pre-fracture 90 %
1 month post-discharge 82 %
(xi) Mean Length stay at Rehabilitation Unit (days): 21.9 ± 6.1
(xii) Patient Destination after hospital discharge: Nursing home 14 %
Family home 86%

SD: standard deviation; MMSE: mini–mental state examination; MNA: mini–nutritional assessment; IQR: interquartile range; FAC: functional ambulation classification.

**Table 2:** Multiple linear regression analysis outcome.

**Dependent variable: functional gain 1 month post- discharge.**				
**(Barthel 1 month- Barthel admission / Barthel prefracture- Barthel admission)**				
**Variable**	**B**	**Standard error**	**p- value**	**95**%** CI**

Age	- 0.014	0.006	0.027	- 0.026, - 0.002

Length of Stay (days)	- 0.012	0.005	0.014	- 0.021, - 0.002

Pre-fracture Barthel	- 0.009	0.02	0.001	- 0.014, - 0.004

Extracapsular Fracture	- 0.157	0.063	0.017	- 0.28, -0.03

MMSE	0.013	0.006	0.045	0.0, 0.026

Albumin serum level	- 0.109	0.062	0.083	- 0.23, 0.015

MMSE: mini–mental state examination.

**Table 3:** Logistic regression analysis outcome. Risk factors predicting worse functional recovery after hip fracture surgery.

**Dependent Variable: 1 month post-discharge FAC**		

Pre-fracture FAC ≥ 4	**p-value**	**OR (95**%** CI)**
	p = 0.039	15.7 (1.14, 215.9)

**Dependent Variable: FAC loss 1 month post-discharge**		

Surgery = Intramedullary Nail	**p-value**	**OR (95**%** CI)**
	p = 0.037	15.66 (1.17, 208.3)

Age (years)	**p-value**	**OR (95**%** CI)**
	p = 0.031	1.58 (1.04, 2.4)

FAC: functional ambulation classification. OR: odds ratio; CI: confidence interval.

## Data Availability

The data used to support the findings of this study are included within the article. Data are also available from the corresponding author upon request.
